# *catena*-Poly[3,3′-diethyl-1,1′-(propane-1,3-di­yl)di(1*H*-imidazol-3-ium) [silver(I)-di-μ-iodido-silver(I)-di-μ-iodido]]

**DOI:** 10.1107/S1600536812020715

**Published:** 2012-05-23

**Authors:** Jian-Zhong Huo, Zhi-Xiang Zhao, Men-Chao Shi, Hui-Long Li, Qing-Xiang Liu

**Affiliations:** a Tianjin Key Laboratory of Structure and Performance for Functional Molecules, College of Chemistry, Tianjin Normal University, Tianjin 300387, People’s Republic of China

## Abstract

The title compound, {(C_13_H_22_N_4_)[Ag_2_I_4_]}_*n*_, was prepared by reaction of 1,3-bis­(*N*-ethyl­imidazolium-1-yl)propane iodide with silver (I) oxide. In the 3,3′-diethyl-1,1′-(propane-1,3-di­yl)di(1*H*-imidazol-3-ium) cation, the dihedral angle between the imidazole rings is 49.3 (1)°. In the [Ag_2_I_4_]^2−^ anion, each Ag^I^ atom is bonded to three iodide anions, the two Ag^I^ atoms and two of the iodides forming Ag_2_I_2_ square-planar (r.m.s. deviation = 0.01 Å) units·The remaining two iodides, which are placed on opposite sides of the square, together with their centrosymmetric counterparts, link the square-planar Ag_2_I_2_ units into {[Ag_2_I_4_]^2−^}_*n*_ polymeric chains *via* Ag—I bonds.

## Related literature

For background to the chemistry of imidazolium compounds, see: Wasserscheid & Keim (2000 ▶); Migowski & Dupont (2007 ▶). For some applications of imidazolium salts, see: Leclercq & Schmitzer (2009 ▶); Petkovic *et al.* (2011 ▶); Chen *et al.* (2006 ▶). For other polymeric chain structures formed *via* Ag—I bonds, see: Chen & Liu (2003 ▶).
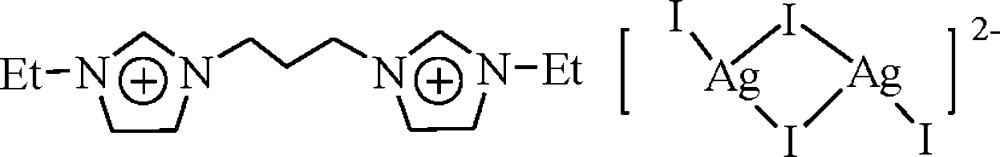


## Experimental

### 

#### Crystal data

(C_13_H_22_N_4_)[Ag_2_I_4_]
*M* *_r_* = 957.69
Triclinic, 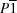

*a* = 9.1202 (18) Å
*b* = 11.543 (2) Å
*c* = 12.158 (2) Å
α = 74.677 (3)°
β = 70.566 (3)°
γ = 79.903 (3)°
*V* = 1158.7 (4) Å^3^
*Z* = 2
Mo *K*α radiation
μ = 7.02 mm^−1^
*T* = 296 K
0.25 × 0.24 × 0.22 mm

#### Data collection

Bruker APEXII CCD area-detector diffractometer
Absorption correction: multi-scan (*SADABS*; Sheldrick, 1996 ▶) *T*_min_ = 0.932, *T*_max_ = 0.987
5868 measured reflections
4008 independent reflections
3692 reflections with *I* > 2σ(*I*)
*R*_int_ = 0.025

#### Refinement

*R*[*F* ^2^ > 2σ(*F* ^2^)] = 0.029
*wR*(*F* ^2^) = 0.075
*S* = 1.04
4008 reflections
211 parameters
H-atom parameters constrained
Δρ_max_ = 1.05 e Å^−3^
Δρ_min_ = −0.86 e Å^−3^

### 

Data collection: *APEX2* (Bruker, 2003 ▶); cell refinement: *SAINT* (Bruker, 2003 ▶); data reduction: *SAINT*; program(s) used to solve structure: *SHELXS97* (Sheldrick, 2008 ▶); program(s) used to refine structure: *SHELXL97* (Sheldrick, 2008 ▶); molecular graphics: *SHELXTL* (Sheldrick, 2008 ▶); software used to prepare material for publication: *SHELXTL*.

## Supplementary Material

Crystal structure: contains datablock(s) global, I. DOI: 10.1107/S1600536812020715/lr2057sup1.cif

Structure factors: contains datablock(s) I. DOI: 10.1107/S1600536812020715/lr2057Isup2.hkl

Additional supplementary materials:  crystallographic information; 3D view; checkCIF report

## Figures and Tables

**Fig. 1.**
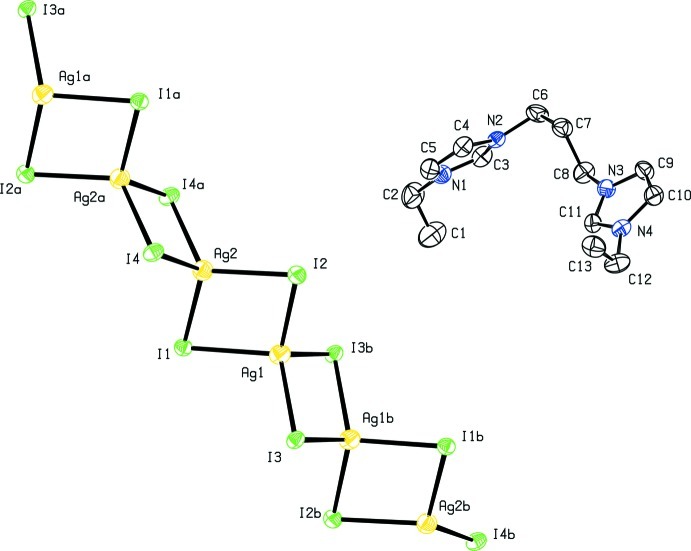
Perspective view of the title compound with anisotropic displacement parameters depicting 30% probability. All hydrogen atoms were omitted for clarity.

## supplementary crystallographic information

### Comment

The design and synthesis of functionalized imidazolium salts are driven by the need for understanding fundamental physical and chemical properties of low melting point salts and modifying them as specific materials (Wasserscheid & Keim, 2000; Migowski & Dupont, 2007). In recent years, the imidazolium salts have been widely studied in ionic liquids (Leclercq & Schmitzer, 2009; Petkovic *et al.*, 2011) and catalytic chemistry (Chen *et al.*, 2006). Herein, we report the preparation and crystal structure of a anionic complex with bis-imidazolium salt, [1,3-bis(*N*-ethylimidazolium-1-yl)propane][Ag_2_I_4_].

The title compound [1,3-dis(*N*-ethylimidazolium-1-yl)propane][Ag_2_I_4_] was prepared *via* the reaction of 1,3-dis(*N*-ethylimidazolium-1-yl)propane iodide with silver oxide (Fig. 1). In the cationic unit of title compound, the dihedral angle between two imidazole rings is 49.3 (1)° (Fig. 2). In the anionic unit [Ag_2_I_4_]^2-^, two silver atoms and two iodine atoms formed a nearly coplanar [Ag_2_I_2_] moiety (the dihedral angle between I1—Ag1—I2 plane and I1—Ag2—I2 plane is 1.2 (5)°), and other two iodine atoms lie in two sides of the [Ag_2_I_2_] plane. Anionic complex [Ag_2_I_4_]^2-^ has been reported, and its formation is strongly influenced by the counteraction. Also, the anionic unit [Ag_2_I_4_]^2-^ can form one-dimensional polymeric chain [Ag_2_I_4_]_n_^2n-^*via* Ag—I bonds (Chen & Liu, 2003). In anion [Ag_2_I_4_]^2-^, each silver atom is surrounded by four iodine atoms to afford a distorted tetrahedral geometry. The I1—Ag1—I2, Ag1—I1—Ag2 and I3—Ag1—I2 bond angles are 101.4 (1)°, 76.8 (2)° and 116.8 (7)°, respectively. The Ag1—I1, Ag1—I2 and Ag1—I3 bond distances are 2.912 (0) Å, 2.908 (9) Å and 2.861 (8) Å, respectively.

### Experimental

A solution of 1-ethylimidazole (1.432 g, 14.9 mmol) and 1,3-diiodopropane (2.000 g, 6.8 mmol) in THF (100 ml) was stirred for three days under refluxing, and a precipitate was formed. The product was filtered and washed with THF, and the white powders of 1, 3-dis(*N*-ethylimidazolium-1-yl)propane iodide was obtained by recrystallization from methanol/diethyl ether. Yield: 2.838 g (86%). Mp: 100–102°C. A suspension of 1, 3-dis(*N*-ethylimidazolium-1-yl)propane iodide (0.200 g, 0.4 mmol) and silver(I) oxide (0.093 g, 0.4 mmol) in dichloromethane (30 ml) was refluxed for 12 h to give a brown solution. The resulting solution was filtered and concentrated to 8 ml, and then diethyl ether (5 ml) was added to precipitate a white powder [1,3-dis(*N*-ethylimidazolium-1-yl)propane][Ag_2_I_4_]. Yield: 0.216 g (55%). Mp: 178–180°C. Anal. Calcd for C_13_H_22_Ag_2_I_4_N_4_: C 16.30, H 2.32, N 5.85%; found: C 16.45, H 2.63, N 5.91%. ^1^H NMR (400 MHz, DMSO-d6): 1.59 (t, J = 7.2 Hz, 6H, CH_3_), 1.76 (m, 2H, CH_2_), 4.62 (q, J = 7.2 Hz, 4H, CH_2_), 5.82 (t, J = 6.6 Hz, 4H, CH_2_), 7.80 (s, 2H, imiH), 7.87 (s, 2H, imiH), 9.46 (s, 2H, 2-imiH) (imi = imidazole).

### Refinement

All H atoms were initially located in a difference Fourier map. They were then placed in geometrically idealized positions and constrained to ride on their parent atoms, with C—H = 0.96 Å(methyl), 0.97 Å (methylene), 0.93 Å (heterocyclic-ring ) and *U*i_so_(H) set to either 1.2 U_eq_(C) or 1.5 U_eq_(C).

### Crystal data

**Table:**

(C_13_H_22_N_4_)[Ag_2_I_4_]	*Z* = 2
*M**_r_* = 957.69	*F*(000) = 868
Triclinic, *P*1	*D*_x_ = 2.745 Mg m^−^^3^
Hall symbol: -P 1	Mo *K*α radiation, λ = 0.71073 Å
*a* = 9.1202 (18) Å	Cell parameters from 5325 reflections
*b* = 11.543 (2) Å	θ = 2.3–27.9°
*c* = 12.158 (2) Å	µ = 7.02 mm^−^^1^
α = 74.677 (3)°	*T* = 296 K
β = 70.566 (3)°	Block, light yellow
γ = 79.903 (3)°	0.25 × 0.24 × 0.22 mm
*V* = 1158.7 (4) Å^3^	

### Data collection

**Table:**

Bruker APEXII CCD area-detector diffractometer	4008 independent reflections
Radiation source: fine-focus sealed tube	3692 reflections with *I* > 2σ(*I*)
Graphite monochromator	*R*_int_ = 0.025
phi and ω scans	θ_max_ = 25.0°, θ_min_ = 1.8°
Absorption correction: multi-scan (*SADABS*; Sheldrick, 1996)	*h* = −9→10
*T*_min_ = 0.932, *T*_max_ = 0.987	*k* = −13→13
5868 measured reflections	*l* = −14→13

### Refinement

**Table:**

Refinement on *F*^2^	Secondary atom site location: difference Fourier map
Least-squares matrix: full	Hydrogen site location: inferred from neighbouring sites
*R*[*F*^2^ > 2σ(*F*^2^)] = 0.029	H-atom parameters constrained
*wR*(*F*^2^) = 0.075	*w* = 1/[σ^2^(*F*_o_^2^) + (0.0283*P*)^2^ + 2.6676*P*] where *P* = (*F*_o_^2^ + 2*F*_c_^2^)/3
*S* = 1.04	(Δ/σ)_max_ = 0.001
4008 reflections	Δρ_max_ = 1.05 e Å^−^^3^
211 parameters	Δρ_min_ = −0.86 e Å^−^^3^
0 restraints	Extinction correction: *SHELXL97* (Sheldrick, 2008), Fc^*^=kFc[1+0.001xFc^2^λ^3^/sin(2θ)]^-1/4^
Primary atom site location: structure-invariant direct methods	Extinction coefficient: 0.00298 (17)

### Special details

**Table:**

Geometry. All e.s.d.'s (except the e.s.d. in the dihedral angle between two l.s. planes) are estimated using the full covariance matrix. The cell e.s.d.'s are taken into account individually in the estimation of e.s.d.'s in distances, angles and torsion angles; correlations between e.s.d.'s in cell parameters are only used when they are defined by crystal symmetry. An approximate (isotropic) treatment of cell e.s.d.'s is used for estimating e.s.d.'s involving l.s. planes.
Refinement. Refinement of *F*^2^ against ALL reflections. The weighted *R*-factor *wR* and goodness of fit *S* are based on *F*^2^, conventional *R*-factors *R* are based on *F*, with *F* set to zero for negative *F*^2^. The threshold expression of *F*^2^ > σ(*F*^2^) is used only for calculating *R*-factors(gt) *etc*. and is not relevant to the choice of reflections for refinement. *R*-factors based on *F*^2^ are statistically about twice as large as those based on *F*, and *R*- factors based on ALL data will be even larger.

### Fractional atomic coordinates and isotropic or equivalent isotropic displacement parameters (Å^2^)

**Table:**

	*x*	*y*	*z*	*U*_iso_*/*U*_eq_	
Ag1	0.49679 (5)	0.38435 (5)	0.12441 (5)	0.06103 (15)	
Ag2	0.51160 (6)	0.13741 (5)	0.37420 (5)	0.06227 (15)	
I1	0.60326 (4)	0.13113 (3)	0.12542 (3)	0.04516 (12)	
I2	0.40256 (4)	0.38216 (3)	0.37820 (3)	0.05260 (12)	
I3	0.75335 (4)	0.51943 (3)	−0.02697 (3)	0.04661 (12)	
I4	0.71765 (4)	0.03787 (3)	0.51764 (3)	0.05221 (13)	
N1	0.0416 (6)	0.6747 (4)	0.6691 (4)	0.0537 (11)	
N2	−0.0555 (5)	0.7953 (4)	0.7900 (4)	0.0409 (9)	
N3	0.0591 (6)	1.1532 (4)	0.7756 (4)	0.0520 (11)	
N4	0.2501 (5)	1.2543 (4)	0.7546 (4)	0.0494 (11)	
C1	0.0810 (13)	0.6785 (8)	0.4606 (7)	0.112 (3)	
H1A	−0.0160	0.7252	0.4568	0.169*	
H1B	0.1122	0.6284	0.4034	0.169*	
H1C	0.1598	0.7318	0.4426	0.169*	
C2	0.0617 (12)	0.6051 (7)	0.5769 (7)	0.091 (3)	
H2A	0.1523	0.5465	0.5756	0.109*	
H2B	−0.0290	0.5611	0.5993	0.109*	
C3	−0.0752 (7)	0.7562 (5)	0.7026 (5)	0.0535 (14)	
H3	−0.1577	0.7820	0.6704	0.064*	
C4	0.0778 (6)	0.7339 (5)	0.8119 (6)	0.0527 (14)	
H4	0.1185	0.7415	0.8701	0.063*	
C5	0.1395 (7)	0.6616 (5)	0.7360 (6)	0.0570 (15)	
H5	0.2323	0.6115	0.7297	0.068*	
C6	−0.1652 (7)	0.8773 (6)	0.8592 (6)	0.0639 (17)	
H6A	−0.2207	0.8311	0.9368	0.077*	
H6B	−0.2414	0.9173	0.8182	0.077*	
C7	−0.0831 (8)	0.9726 (6)	0.8771 (6)	0.0601 (15)	
H7A	−0.1545	1.0129	0.9378	0.072*	
H7B	0.0059	0.9340	0.9042	0.072*	
C8	−0.0296 (8)	1.0630 (6)	0.7621 (6)	0.0629 (16)	
H8A	−0.1194	1.1045	0.7374	0.076*	
H8B	0.0367	1.0218	0.7004	0.076*	
C9	−0.0017 (7)	1.2409 (5)	0.8407 (5)	0.0556 (14)	
H9	−0.1052	1.2541	0.8859	0.067*	
C10	0.1163 (7)	1.3039 (5)	0.8267 (5)	0.0576 (15)	
H10	0.1090	1.3694	0.8598	0.069*	
C11	0.2117 (7)	1.1627 (5)	0.7253 (5)	0.0525 (13)	
H11	0.2805	1.1133	0.6774	0.063*	
C12	0.4114 (8)	1.2921 (7)	0.7163 (6)	0.0737 (19)	
H12A	0.4710	1.2680	0.6421	0.088*	
H12B	0.4052	1.3794	0.7009	0.088*	
C13	0.4953 (7)	1.2391 (7)	0.8069 (7)	0.0719 (19)	
H13A	0.4354	1.2606	0.8814	0.108*	
H13B	0.5956	1.2697	0.7797	0.108*	
H13C	0.5089	1.1529	0.8181	0.108*	

### Atomic displacement parameters (Å^2^)

**Table:**

	*U*^11^	*U*^22^	*U*^33^	*U*^12^	*U*^13^	*U*^23^
Ag1	0.0493 (3)	0.0654 (3)	0.0668 (3)	−0.0107 (2)	−0.0207 (2)	−0.0044 (2)
Ag2	0.0636 (3)	0.0600 (3)	0.0629 (3)	−0.0068 (2)	−0.0189 (2)	−0.0128 (2)
I1	0.0412 (2)	0.0478 (2)	0.0471 (2)	−0.00823 (15)	−0.01187 (15)	−0.01073 (15)
I2	0.0532 (2)	0.0519 (2)	0.0513 (2)	−0.00460 (17)	−0.01208 (17)	−0.01417 (17)
I3	0.03871 (19)	0.0443 (2)	0.0542 (2)	−0.00964 (14)	−0.01653 (15)	0.00034 (16)
I4	0.0522 (2)	0.0540 (2)	0.0524 (2)	−0.01805 (17)	−0.01913 (17)	−0.00228 (17)
N1	0.059 (3)	0.051 (3)	0.051 (3)	−0.009 (2)	−0.016 (2)	−0.010 (2)
N2	0.033 (2)	0.043 (2)	0.048 (2)	−0.0121 (18)	−0.0158 (18)	−0.0016 (19)
N3	0.058 (3)	0.045 (2)	0.054 (3)	−0.011 (2)	−0.010 (2)	−0.017 (2)
N4	0.057 (3)	0.047 (2)	0.045 (3)	−0.014 (2)	−0.017 (2)	−0.004 (2)
C1	0.199 (11)	0.081 (6)	0.066 (5)	−0.043 (6)	−0.034 (6)	−0.019 (4)
C2	0.138 (8)	0.065 (4)	0.083 (5)	−0.014 (5)	−0.037 (5)	−0.027 (4)
C3	0.057 (3)	0.056 (3)	0.059 (3)	−0.012 (3)	−0.035 (3)	−0.004 (3)
C4	0.044 (3)	0.054 (3)	0.067 (4)	−0.011 (3)	−0.031 (3)	−0.002 (3)
C5	0.039 (3)	0.053 (3)	0.080 (4)	−0.008 (3)	−0.021 (3)	−0.010 (3)
C6	0.042 (3)	0.074 (4)	0.070 (4)	−0.021 (3)	0.003 (3)	−0.021 (3)
C7	0.063 (4)	0.064 (4)	0.054 (3)	−0.009 (3)	−0.011 (3)	−0.021 (3)
C8	0.075 (4)	0.060 (4)	0.063 (4)	−0.016 (3)	−0.027 (3)	−0.014 (3)
C9	0.051 (3)	0.057 (3)	0.059 (3)	−0.005 (3)	−0.009 (3)	−0.024 (3)
C10	0.071 (4)	0.052 (3)	0.055 (3)	−0.009 (3)	−0.018 (3)	−0.019 (3)
C11	0.058 (3)	0.044 (3)	0.053 (3)	−0.001 (3)	−0.014 (3)	−0.013 (3)
C12	0.073 (5)	0.084 (5)	0.064 (4)	−0.034 (4)	−0.019 (3)	−0.002 (4)
C13	0.050 (4)	0.093 (5)	0.074 (4)	−0.005 (3)	−0.023 (3)	−0.018 (4)

### Geometric parameters (Å, º)

**Table:**

Ag1—I3^i^	2.8383 (7)	C1—H1C	0.9600
Ag1—I3	2.8618 (7)	C2—H2A	0.9700
Ag1—I2	2.9089 (9)	C2—H2B	0.9700
Ag1—I1	2.9120 (8)	C3—H3	0.9300
Ag2—I2	2.8333 (8)	C4—C5	1.327 (9)
Ag2—I4	2.8735 (7)	C4—H4	0.9300
Ag2—I1	2.8749 (8)	C5—H5	0.9300
Ag2—I4^ii^	2.9054 (8)	C6—C7	1.529 (8)
I3—Ag1^i^	2.8383 (7)	C6—H6A	0.9700
I4—Ag2^ii^	2.9054 (7)	C6—H6B	0.9700
N1—C3	1.322 (8)	C7—C8	1.495 (9)
N1—C5	1.362 (7)	C7—H7A	0.9700
N1—C2	1.491 (8)	C7—H7B	0.9700
N2—C3	1.332 (7)	C8—H8A	0.9700
N2—C4	1.366 (7)	C8—H8B	0.9700
N2—C6	1.460 (7)	C9—C10	1.342 (8)
N3—C11	1.332 (7)	C9—H9	0.9300
N3—C9	1.376 (7)	C10—H10	0.9300
N3—C8	1.496 (7)	C11—H11	0.9300
N4—C11	1.333 (7)	C12—C13	1.488 (10)
N4—C10	1.376 (7)	C12—H12A	0.9700
N4—C12	1.495 (8)	C12—H12B	0.9700
C1—C2	1.415 (11)	C13—H13A	0.9600
C1—H1A	0.9600	C13—H13B	0.9600
C1—H1B	0.9600	C13—H13C	0.9600
			
I3^i^—Ag1—I3	105.33 (2)	C5—C4—H4	125.8
I3^i^—Ag1—I2	111.08 (2)	N2—C4—H4	125.8
I3—Ag1—I2	116.87 (2)	C4—C5—N1	107.1 (5)
I3^i^—Ag1—I1	115.75 (2)	C4—C5—H5	126.4
I3—Ag1—I1	106.74 (2)	N1—C5—H5	126.4
I2—Ag1—I1	101.418 (18)	N2—C6—C7	112.2 (5)
I2—Ag2—I4	112.83 (2)	N2—C6—H6A	109.2
I2—Ag2—I1	104.221 (19)	C7—C6—H6A	109.2
I4—Ag2—I1	121.64 (2)	N2—C6—H6B	109.2
I2—Ag2—I4^ii^	117.25 (2)	C7—C6—H6B	109.2
I4—Ag2—I4^ii^	98.81 (2)	H6A—C6—H6B	107.9
I1—Ag2—I4^ii^	102.35 (2)	C8—C7—C6	110.0 (5)
Ag2—I1—Ag1	76.825 (17)	C8—C7—H7A	109.7
Ag2—I2—Ag1	77.525 (17)	C6—C7—H7A	109.7
Ag1^i^—I3—Ag1	74.67 (2)	C8—C7—H7B	109.7
Ag2—I4—Ag2^ii^	81.19 (2)	C6—C7—H7B	109.7
C3—N1—C5	108.4 (5)	H7A—C7—H7B	108.2
C3—N1—C2	126.4 (6)	C7—C8—N3	111.0 (5)
C5—N1—C2	125.2 (6)	C7—C8—H8A	109.4
C3—N2—C4	107.0 (5)	N3—C8—H8A	109.4
C3—N2—C6	126.4 (5)	C7—C8—H8B	109.4
C4—N2—C6	126.1 (5)	N3—C8—H8B	109.4
C11—N3—C9	108.1 (5)	H8A—C8—H8B	108.0
C11—N3—C8	125.4 (5)	C10—C9—N3	107.2 (5)
C9—N3—C8	126.4 (5)	C10—C9—H9	126.4
C11—N4—C10	107.7 (5)	N3—C9—H9	126.4
C11—N4—C12	124.9 (5)	C9—C10—N4	107.9 (5)
C10—N4—C12	127.4 (5)	C9—C10—H10	126.1
C2—C1—H1A	109.5	N4—C10—H10	126.1
C2—C1—H1B	109.5	N3—C11—N4	109.1 (5)
H1A—C1—H1B	109.5	N3—C11—H11	125.4
C2—C1—H1C	109.5	N4—C11—H11	125.4
H1A—C1—H1C	109.5	C13—C12—N4	113.0 (6)
H1B—C1—H1C	109.5	C13—C12—H12A	109.0
C1—C2—N1	113.4 (6)	N4—C12—H12A	109.0
C1—C2—H2A	108.9	C13—C12—H12B	109.0
N1—C2—H2A	108.9	N4—C12—H12B	109.0
C1—C2—H2B	108.9	H12A—C12—H12B	107.8
N1—C2—H2B	108.9	C12—C13—H13A	109.5
H2A—C2—H2B	107.7	C12—C13—H13B	109.5
N1—C3—N2	109.0 (5)	H13A—C13—H13B	109.5
N1—C3—H3	125.5	C12—C13—H13C	109.5
N2—C3—H3	125.5	H13A—C13—H13C	109.5
C5—C4—N2	108.4 (5)	H13B—C13—H13C	109.5
			
I2—Ag2—I1—Ag1	0.813 (16)	C6—N2—C3—N1	−174.1 (5)
I4—Ag2—I1—Ag1	−127.96 (2)	C3—N2—C4—C5	1.8 (6)
I4^ii^—Ag2—I1—Ag1	123.43 (2)	C6—N2—C4—C5	175.1 (5)
I3^i^—Ag1—I1—Ag2	−121.10 (2)	N2—C4—C5—N1	−2.1 (7)
I3—Ag1—I1—Ag2	122.05 (2)	C3—N1—C5—C4	1.6 (7)
I2—Ag1—I1—Ag2	−0.783 (16)	C2—N1—C5—C4	−176.6 (6)
I4—Ag2—I2—Ag1	133.12 (2)	C3—N2—C6—C7	−137.1 (6)
I1—Ag2—I2—Ag1	−0.811 (16)	C4—N2—C6—C7	50.9 (8)
I4^ii^—Ag2—I2—Ag1	−113.07 (2)	N2—C6—C7—C8	72.4 (7)
I3^i^—Ag1—I2—Ag2	124.36 (2)	C6—C7—C8—N3	−176.8 (5)
I3—Ag1—I2—Ag2	−114.78 (3)	C11—N3—C8—C7	111.0 (7)
I1—Ag1—I2—Ag2	0.792 (16)	C9—N3—C8—C7	−69.5 (8)
I3^i^—Ag1—I3—Ag1^i^	0.0	C11—N3—C9—C10	1.0 (7)
I2—Ag1—I3—Ag1^i^	−123.85 (3)	C8—N3—C9—C10	−178.5 (6)
I1—Ag1—I3—Ag1^i^	123.56 (3)	N3—C9—C10—N4	−0.8 (7)
I2—Ag2—I4—Ag2^ii^	124.61 (3)	C11—N4—C10—C9	0.3 (7)
I1—Ag2—I4—Ag2^ii^	−110.47 (3)	C12—N4—C10—C9	−178.5 (6)
I4^ii^—Ag2—I4—Ag2^ii^	0.0	C9—N3—C11—N4	−0.8 (7)
C3—N1—C2—C1	63.8 (11)	C8—N3—C11—N4	178.7 (5)
C5—N1—C2—C1	−118.4 (9)	C10—N4—C11—N3	0.3 (6)
C5—N1—C3—N2	−0.4 (6)	C12—N4—C11—N3	179.2 (5)
C2—N1—C3—N2	177.7 (6)	C11—N4—C12—C13	−95.5 (8)
C4—N2—C3—N1	−0.8 (6)	C10—N4—C12—C13	83.2 (8)

Symmetry codes: (i) −*x*+1, −*y*+1, −*z*; (ii) −*x*+1, −*y*, −*z*+1.
